# Cave-Dwelling Fish Provide Clues to the Circadian Cycle

**DOI:** 10.1371/journal.pbio.1001141

**Published:** 2011-09-06

**Authors:** Robin Mejia

**Affiliations:** Freelance Science Writer, Albany, California, United States of America

**Figure:**
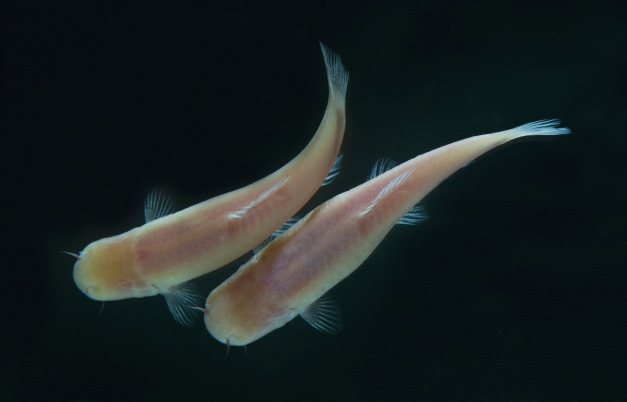
The Somalian cavefish *Phreatichthys andruzzii* were collected in the wild at the oasis of Bud-Bud in the center of the Somalian desert. Image Credit: Saulo Bambi.

Whether it's a jay that starts its day at first light, an ice worm that burrows into a glacier when the sun comes out, or a bat that heads out to feed at dusk, virtually all animals follow a daily pattern of activity. While the need for this kind of pattern, called the circadian rhythm, is not fully understood, it is remarkably conserved across species, and disruptions to an animal's circadian cycle produce stress. In humans, such disruptions are associated with insomnia and depression.

As in most mammals, the timing of the circadian clock appears to be in part maintained by exposure to daylight. Cells in the retina react to light, sending signals to a “master” time keeper in the hypothalamus. That's why light exposure helps us recover from jet lag; the daylight patterns of a new time zone eventually reset the body's clock. Daylight also appears to help maintain regular rhythms. In experiments where people are kept in constant dimness, they can maintain fairly regular sleep/wake cycles, but their “days” (or so-called period) expand slightly beyond 24 hours, eventually moving out of sync with the outside world. Although researchers have identified several genes involved in keeping the circadian clock running, the entire process is not completely understood. In this issue of *PLoS Biology*, Cavallari and colleagues use a standard circadian model, the zebrafish, to examine how two different photoreceptors help set the circadian clock.

In addition to possessing a central clock mechanism in the brain, in other tissues zebrafish cells are known to possess “peripheral” clock mechanisms that are regulated directly by sunlight. This suggests that specific photoreceptors are involved in regulating the peripheral clocks, but it's not clear which ones. To analyze these clocks, the researchers decided to compare the zebrafish to a species of fish that had evolved without sunlight for between 1.4 and 2.6 million years. They chose the Somalian cavefish, *Phreatichthys andruzzii*, because it has been isolated from the day-night cycle for approximately 1 million years longer than more well-studied cavefish species. Having lived without sunlight for so many generations, *P. andruzzii* no longer has any eye function or scales. But does loss of vision also mean loss of peripheral circadian clock regulation by light?

To assess whether *P. andruzzii* still possessed a light-responsive circadian clock, Cavallari and colleagues first exposed the fish to alternating 12-hour cycles of light and darkness. They found that the cavefish was active at irregular periods that did not relate to the light, whereas zebrafish kept under the same conditions exhibited a standard diurnal pattern. Next, the researchers measured the activity of cavefish genes that are homologs of known clock genes in the zebrafish. Measuring gene activity in adult tissue and whole larvae, once again, the scientists found that while the zebrafish genes responded to light, the cavefish genes did not.

But light may not be the only regulator of circadian clocks in these species. While daylight is the predominant clock regulator, animal clocks are known to respond to other stimuli, including food. So, for one month, researchers fed the fish at the same time each day. Both fish exhibited food anticipatory behavior, a sign of clock action. At the end of the month, researchers tested for gene activity. This time, they found clock action in both types of fish; in zebrafish they found responses to both light and food signals, while in cavefish they observed only a response to the food.

The researchers next wanted to examine what part of the light-driven clock had been disrupted in the cavefish. First, they determined that the clock genes themselves were normal, as were the genes that directly regulate the clock genes. So they turned to the photoreceptors whose initial reaction to sunlight starts the process, choosing two well-known receptors as likely candidates: melanopsin and TMT-opsin.

When they examined the cavefish version of those two genes, they found truncation mutations that would cause translation of the protein to stop early, completely eliminating key regions of the molecule known to be involved in the light response. To confirm that the photoreceptors were the problem, they introduced zebrafish genes for melanopsin and TMT-opsin into cavefish cell lines. The transgenic cavefish cells' clock began responding to light, providing direct evidence that those two photoreceptors play a role in light-induced regulation of the circadian clock.

This is not the end of the story, however. Zebrafish cells respond to red, green, and blue light. The transgenic cavefish cells only respond to blue and green light, indicating that there are additional photoreceptors in the zebrafish that have yet to be discovered. Furthermore, when cavefish cells were exposed to glucocorticoids known to reset the clock, they reacted, exhibiting a clock period of 43 hours, much longer than is seen in most light-responsive animals even when they are removed from light exposure.

By demonstrating the value of a cavefish model that has evolved for millions of years in darkness, Cavallari and colleagues have shown that this species still retains a circadian clock, and that mutations in two specific photoreceptors have led to the loss of a light-entrainable clock. This work highlights the utility of the cavefish for studying the evolution and regulation of the circadian clock for future research.

**Cavallari N, Frigato E, Vallone D, Fröhlich N, Lopez-Olmeda JF, et al. (2011) A Blind Circadian Clock in Cavefish Reveals that Opsins Mediate Peripheral Clock Photoreception. doi:10.1371/journal.pbio.1001142**

